# Changes in vitamins and trace elements after initiation of highly effective CFTR modulator therapy in children and adults with cystic fibrosis – a real-life insight

**DOI:** 10.1186/s40348-024-00178-6

**Published:** 2024-05-08

**Authors:** Dorit Fabricius, Tina Knieling, Noelle Zurmuehl, Leandra Makedon, Joachim Freihorst, Hanna Schmidt, Sebastian Bode

**Affiliations:** Department of Pediatrics and Adolescent Medicine, Ulm University Medical Center, Ulm University, Eythstrasse 24, 89075 Ulm, Germany

**Keywords:** Cystic fibrosis, Vitamins, Trace elements, Micronutrients, Highly effective CFTR modulator therapy, Individualized therapy

## Abstract

**Background:**

Highly-effective CFTR-modulator therapy with elexa-/teza-/ivacaftor (ETI) has led to improvements in pulmonary outcomes, sweat chloride, body mass index (BMI) and quality of life in people with cystic fibrosis (CF). Improved uptake of fat-soluble vitamins and micronutrients has been reported for CFTR-modulators but data regarding ETI therapy is lacking.

**Methods:**

This single-center retrospective study evaluated forced expiratory volume in one second (FEV-1), sweat chloride, BMI, transaminases (AST, ALT), bilirubin, vitamins A, D, E, zinc and selenium in children and adults eligible for ETI. Parameters were assessed before and up to one year after initiation of ETI.

**Results:**

58 patients (median age m = 28 years, SD ± 11.6 years, 51.7% female14 < 18 years old) were included. FEV-1 and sweat chloride improved significantly after ETI. There were no changes in BMI or AST. ALT was increased significantly after 4 weeks of ETI but returned to normal levels in further course. Bilirubin levels remained elevated after ETI. Vitamin A was significantly higher 12 months after ETI. No changes were found for vitamins D, E, zinc and selenium.

**Conclusions:**

This study adds to the evidence that improvements of some fat-soluble vitamin levels can be found after ETI. No changes regarding micronutrients were noted. Individualized follow-up and supplementation are recommended.

## Background

Cystic fibrosis (CF) is a multi-system condition caused by mutations in the *cystic fibrosis transmembrane conductance regulator* (CFTR) gene that cause impaired production or functioning of the CFTR-protein which is essential for chloride and bicarbonate transport across epithelial cell membranes [1]. Mal- or nonfunctioning CFTR leads to pathologic viscosity of secretions in the lungs, pancreas, intestines, and reproductive organs. Besides pulmonary pathology, cystic fibrosis is characterized by pancreatic insufficiency in many patients and the majority of patients show signs of pancreatic insufficiency already at birth [2, 3]. Intestinal inflammation and gastrointestinal symptoms often cause impaired appetite contributing to poor weight gain [4]. Both pancreatic insufficiency and persistent intestinal inflammation have been reported to cause reduced intestinal absorption of fat and fat-soluble vitamins (vitamins A, D, E, and K) as well as trace elements as zinc and selenium in CF [5]. Pancreatic enzyme replacement therapy (PERT) is a cornerstone of treatment of pancreatic insufficiency in CF. Pancreatic lipase containing formulations have to be administered with every meal, depending on its fat content. A high-calorie, high-protein and high-fat diet has been traditionally recommended for people with CF. Fat-soluble vitamins need to be supplemented as most patients with CF and PERT alone develop vitamin deficiencies and the levels of fat-soluble vitamins need to be monitored regularly [6, 7]. Less data exist regarding micronutrients such as zinc and selenium but these trace elements are supplemented by many patients with CF [8].

The development of CFTR-modulators, a group of small molecules that includes CFTR-correctors and CFTR-potentiators, has been a breakthrough for many people with CF [9]. Depending on the genotype, different CFTR-modulators are indicated. Improvement of both intestinal inflammation and in some cases even in pancreatic function have been reported for lumacaftor/ivacaftor [10–12]. Highly-effective CFTR-modulator therapy, containing the correctors elexacaftor and tezacaftor as well as the potentiator ivacaftor (ETI), has shown dramatic positive effects on pulmonary function, reduction of pulmonary exacerbations, body mass index (BMI) and quality of life [13–15]. ETI is approved in people with cystic fibrosis with one Phe508del plus a minimal or residual function mutation in *CFTR* (at the moment in individuals ≥ 2 years in Europe). A temporary increase in transaminases and bilirubin is well known after start of ETI therapy [13, 15, 16]. ETI therapy also seems to influence intestinal uptake of nutrients, including vitamins and trace elements. Individuals with cystic fibrosis for the first time face new challenges regarding nutrition [17] and many traditional treatment concepts, including fat-soluble vitamin and micronutrient supplementation should be evaluated. Some data exist regarding fat-soluble vitamins and trace elements in people with CF who were treated with CFTR-modulators:

Vitamin A is crucial for antioxidative functions, immunity, cell proliferation as well as differentiation of cells [18]. Normal vitamin A levels are associated with better pulmonary outcomes in CF [19]. Vitamin A deficiency was becoming rare in CF even before CFTR-modulator therapy and elevated serum vitamin A levels were more frequently observed [20]. After initiation of lumacaftor/ivacaftor higher vitamin A levels have been reported [21, 22]. Both improved vitamin A levels [23] but also case reports of vitamin A intoxication after start of ETI have been published [24, 25].

Vitamin D is essential for bone metabolism and plays important roles in immunity, microbiome and pulmonary health [18]. Data exist regarding better pulmonary function in individuals with CF and optimal vitamin D levels but a meta-analysis could not confirm these findings [26]. In rare cases vitamin D toxicity has been reported in CF-patients even in the pre-CFTR-modulator era and these were mainly caused by dosing errors while preparing individualized medications [27]. Both luma-/ivacaftor and ETI therapy have been shown to improve vitamin D levels [21, 23, 28].

Vitamin E has antioxidant functions. There is no clear link between optimal vitamin E levels and health in CF [29]. Higher levels of Vitamin E have been shown after therapy with luma-/ivacaftor [21, 30] and with ETI treatment [23].

Vitamin K is best known for its importance in synthetization of clotting factors II, VII, IX, and X. Many individuals with CF have vitamin K deficiency but detrimental effects are rare [31]. Effects of overdosing or changes after CFTR-modulator therapy have not been described [18].

Zinc is a trace element with metabolic, antioxidative and anti-inflammatory properties. Zinc deficiency has been associated with failure to thrive, reduced pulmonary function, bone disease and impaired glucose tolerance in CF [32]. Zinc supplementation might help reduce pulmonary exacerbations in children with CF [33]. There are no data on zinc levels under ETI therapy.

Selenium is also a trace element with antioxidative, anti-inflammatory and metabolic functions. An increase in selenium levels was noticed after 10 months of treatment with lumacaftor/Ivacaftor [30]. It is unclear though if an optimal selenium level exists and supplementation of selenium is not associated with better patient outcomes in CF [8]. No data on selenium levels after ETI therapy have been published.

Even though changes in fat-soluble vitamins and in trace element levels have been found after onset of CFTR-modulator therapy there still is a lack of data regarding these changes after initiation of highly-effective CFTR-modulator therapy with ETI in a larger cohort and especially in children with CF. The aim of our study was to provide real-life insights of changes in fat-soluble vitamin and trace element concentrations in children and adults with CF, homozygous or heterozygous for Phe508del, who started ETI therapy. Subjects have been followed up for at least 12 months.

## Methods

### Patient identification

Patients who were treated at our outpatient CF-clinic were screened for their eligibility for ETI-treatment according to their respective CFTR-mutations and according to European approval of ETI. Patients were informed about ETI therapy including potential benefits and possible adverse effects. If patients consented, therapy with ETI was started. ETI therefore was part of patients’ routine care and this is an observational retrospective study. This study included patients who started ETI therapy between August 21st, 2020 and March 1st, 2022, therefore mainly adults and some children < 18 years were included. All patients were screened regarding their liver function before initiation of ETI therapy. All patients with pre-existing CF-hepatopathy or increased transaminases or bilirubin received ursodeoxycholic acid. All persons with CF undergo regular routine laboratory work at our institution and laboratory parameters done about 12 months and four weeks before start of ETI were included in the analysis. Blood collections regarding liver function parameters (ALT, AST, bilirubin) were performed 4 weeks after start of ETI and three, six, nine andl 12 months after beginning ETI therapy. Additional laboratory work was performed in exacerbations or other patient-dependent individual circumstances. All laboratory work was part of patients’ routine care and done at the treating physician’s discretion. At our institution subjects with CF undergo yearly extensive additional laboratory testing including evaluation of vitamins A, D, E and trace elements (zinc and selenium) as suggested [34]. These appointments occurred at least once before and twice after initiation of ETI in the time period reported. Time intervals to therapy initiation varied across the cohort. Not all patients had laboratory work-up performed at all time-points and missing data was not an exclusion criterium for this study. Sweat chloride was measured before initiation of ETI and three months afterwards. Pulmonary function tests were performed according to global lung initiative/European Respiratory Society guidelines [35] at least every three months and additionally four weeks after start of ETI. Exclusion criteria included genotype not eligible for ETI treatment, age < 6 years, poor pulmonary function (forced expiratory volume in one second in percent predicted (FEV-1%pred) < 40%, liver cirrhosis, pancreatic sufficiency (fecal elastase > 200 µg/g stool) and known poor adherence to medication. If patients experienced an acute exacerbation, any laboratory results obtained at the same time were excluded from the study.

### Supplementation, laboratory work-up & pulmonary function tests

Supplementation of vitamins and trace elements was conducted according to current guidelines [36]. All patients received combination preparations including vitamin A, vitamin D, vitamin E, vitamin K, zinc, and selenium. Patients were questioned regarding additional vitamin supplementation (e.g. prescribed by their primary physicians or bought over the counter). If amounts of vitamins taken were deemed acceptable by the treating physician’s respective laboratory values were not excluded. All patients took PERT. Levels of vitamins A, D and E as well as zinc and selenium were measured at the central laboratory services of our University Hospital. Vitamin K measurement was locally not available and therefore is not included in routine laboratory check-ups. We decided against including coagulation measures as surrogate parameters as no changes have been reported after CFTR-modulator therapy at least for lumacaftor/ivacaftor [22]. Vitamins A and E were measured via high-performance liquid chromatography (Chromsystems Instruments & Chemicals GmbH, Graefelfing, Germany), Vitamin D via electro-chemi-luminescense-immunoassay (Roche, Basel, Switzerland). ALT, AST, total bilirubin and zinc were measured photometrically on the Cobas C system (Roche, Basel, Switzerland) and selenium by graphite tube atomic absorption spectroscopy. Sweat chloride measurements were performed on the FKGO chloridemeter 20 (Kreienbaum, Langenfeld, Germany). Pulmonary function tests were performed according to European Respiratory Society (ERS) Guidelines and published reference values for FEV-1 were used [35, 37]. Pulmonary function tests were performed with the “Master Screen Body” (Vyaire Medical GmbH, Höchberg, Germany).

### Ethics

#### Ethics approval

was obtained from the Ulm University ethics committee (permit number 228/21, April 5th, 2022). As this was a retrospective study the need for additional informed consent was waived by the ethics committee.

### Data analysis and statistics

The statistical analysis was conducted using GraphPad Prism (Version 7.01, GraphPad Software, La Jolla, CA, USA, www.graphpad.com). Categorical data were presented as means and standard deviations. For temporal differences, t-tests for pairwise differences and sign rank tests were utilized based on the distribution of the data. The significance level was set at *p* < .05.

## Results

Between August 21st, 2020 and March 1st, 2022, 58 patients (30 female, 28 male) started ETI therapy at our institution and could be followed up for at least 12 months. Patients were 7–59 years old (m = 28.7 years, SD ± 11.6 years) (Table 1). 8 patients had been treated with lumacaftor/ivacaftor before. FEV-1 increased significantly from 2.3 l (64%pred) before ETI to 2.69 l (75.6%pred) 12 months after ETI (*p* < .001). BMI was 21.88 kg/m^2^ before and 21.59 kg/m^2^ after 12 months of therapy with no significant change (*p* = .65) (Table 2).

**Table 1:** Demographic data of the cohort. n = number

	number	Female: *n* (%)	Phe508del homozygous: *n* (%)	Phe508del heterozygous: *n* (%)
Total cohort	58	30 (51.7)	32 (55.2)	26 (44.8)
> 18 years	44	25 (56.8)	23 (52.3)	21 (47.7)
12–18 years	13	5 (38.4)	9 (69.2)	4 (30.8)
6–12 years	1	0 (0)	0 (0)	1 (100)

**Table 2:** Means and standard deviations of body mass index (BMI) and forced expiratory volume in 1 s (FEV-1) before and after ETI therapy. SD = standard deviation

	BMI [kg/m^2^], mean (± SD)			FEV-1: [%pred], mean (± SD)		
	4 weeks pre	12 months post	*p*-value pre/post	4 weeks pre	12 months post	*p*-value pre/post
Total cohort	21.88 (± 4.19)	21.96 (± 3.03)	n.s.	64.14 (± 25.68)	75.57 (± 26.53)	**< 0.001**
> 18 years	22.52 (± 4.17)	22.43 (± 2.67)	n.s.	56.77 (± 21.35)	66.63 (± 22.74)	**< 0.001**
12–18 years	20.24 (± 3.55)	20.42 (± 3.59)	n.s.	87.38 (± 24.29)	103.08 (± 16.57)	**0.03**
6–12 years	15	15	n.s.	90	89	n.s.

**Table 3:** Means and standard deviations of AST, ALT, total bilirubin, vitamin A, vitamin D, vitamin E, zinc, selenium, and sweat chloride before and after initiation of ETI therapy. Vitamins and trace elements were not evaluated four weeks after start of ETI therefore values are missing. Values are represented in bold print if the change was significant (*p* < .05) compared to 4 weeks prior to ETI therapy and in italic print if significantly different to 12 months before ETI. ALT = alanine aminotransferase, AST = aspartate aminotransferase

Laboratory value [unit] (normal range)	12 months before start	4 weeks before start	4 weeks after start	3 months after start	6 months after start	9 months after start	12 months after start
**AST [U/l] (< 35)**	30.68 ± 14.91	29.04 ± 11.6	35.15 ± 18.56	29.74 ± 12.26	31.85 ± 18.79	32.39 ± 14.91	30.75 ± 16.24
**ALT [U/l] (< 34)**	30.49 ± 32.09	26.18 ± 15.55	**38.35 ± 24.25**	31.66 ± 21.47	36.94 ± 39.41	33.22 ± 26.39	35.09 ± 48.00
**total bilirubin [µmol/l] (2–21)**	8.02 ± 8.68	7.32 ± 7.61	***14.89 ± 13.61***	***13.93 ± 12.77***	***14.23 ± 14.97***	***11.74 ± 7.73***	***14.19 ± 10.49***
**vitamin A [µmol/l] (1.05–2.8)**	1.37 ± 0.41	1.41 ± 0.64		1.57 ± 0.53	1.47 ± 0.39	1.53 ± 0.39	***1.74 ± 0.38***
**vitamin D [µg/l] (20–50)**	26.76 ± 10.52	27.26 ± 9.05	29.33 ± 9.29	25.36 ± 8.70	25.07 ± 9.00	31.06 ± 8.64	28.23 ± 17.78
**vitamin E [µmol/l] (12–42)**	23.15 ± 7.33	24.02 ± 8.51		25.81 ± 10.4	25.72 ± 8.68	27.88 ± 9.24	28.6 ± 11.85
**zinc [µmol/l] (10.7–18.4)**	10.87 ± 1.56	13.77 ± 2.56		11.94 ± 2.48	11.15 ± 1.90	12.38 ± 2.90	13.5 ± 2.16
**selenium [µg/l] (60–130)**	108.7 ± 52.31	224 ± 187.5	442.80 ± 396.30	117.90 ± 79.90	269.50 ± 295.30	190.50 ± 115.70	164.80 ± 170.60
**Sweat chloride [mmol/l] (< 30)**	87.67 ± 22.37	101.70 ± 25.20	56.67 ± 20.21	**63.25 ± 24.11**	**55.81 ± 25.52**	**49.30 ± 8.20**	62.5 ± 32.51

**Fig. 1:**
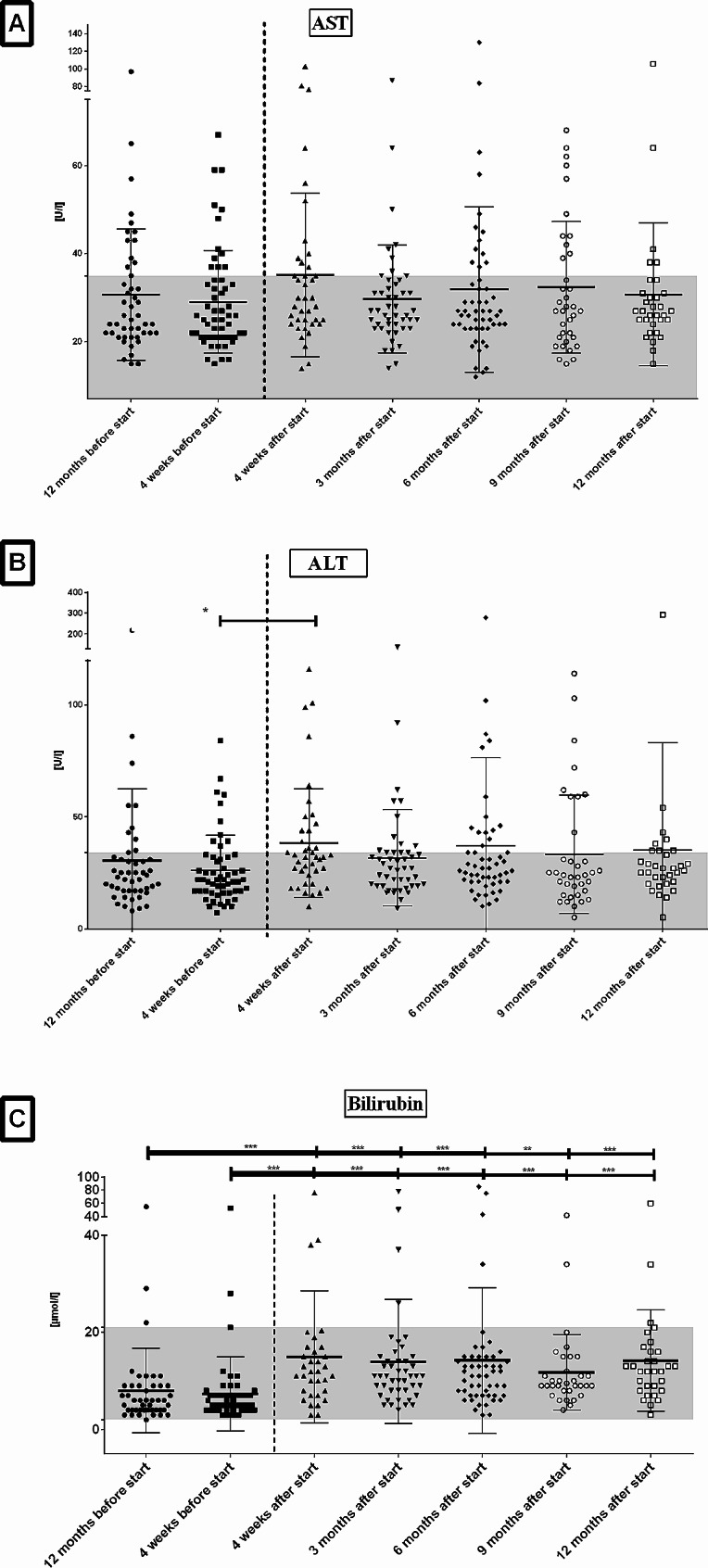
**A**: No changes in aspartate-aminotransferase levels before and up to 12 months after start of ETI. **B**: Significant change of alanine-aminotransferase four weeks after initiation of ETI, no other changes. **C**: Significant and persistent elevation of total bilirubin levels after start of ETI at all timepoints compared to before start of ETI therapy. Reference ranges of AST (< 35 U/l), ALT (< 34 U/l) and bilirubin (2–21 µmol/l) are indicated in grey. AST = aspartate-aminotransferase, ALT = alanine-aminotransferase, ETI = elexa-/teza-/ivacaftor.

**Fig. 2:**
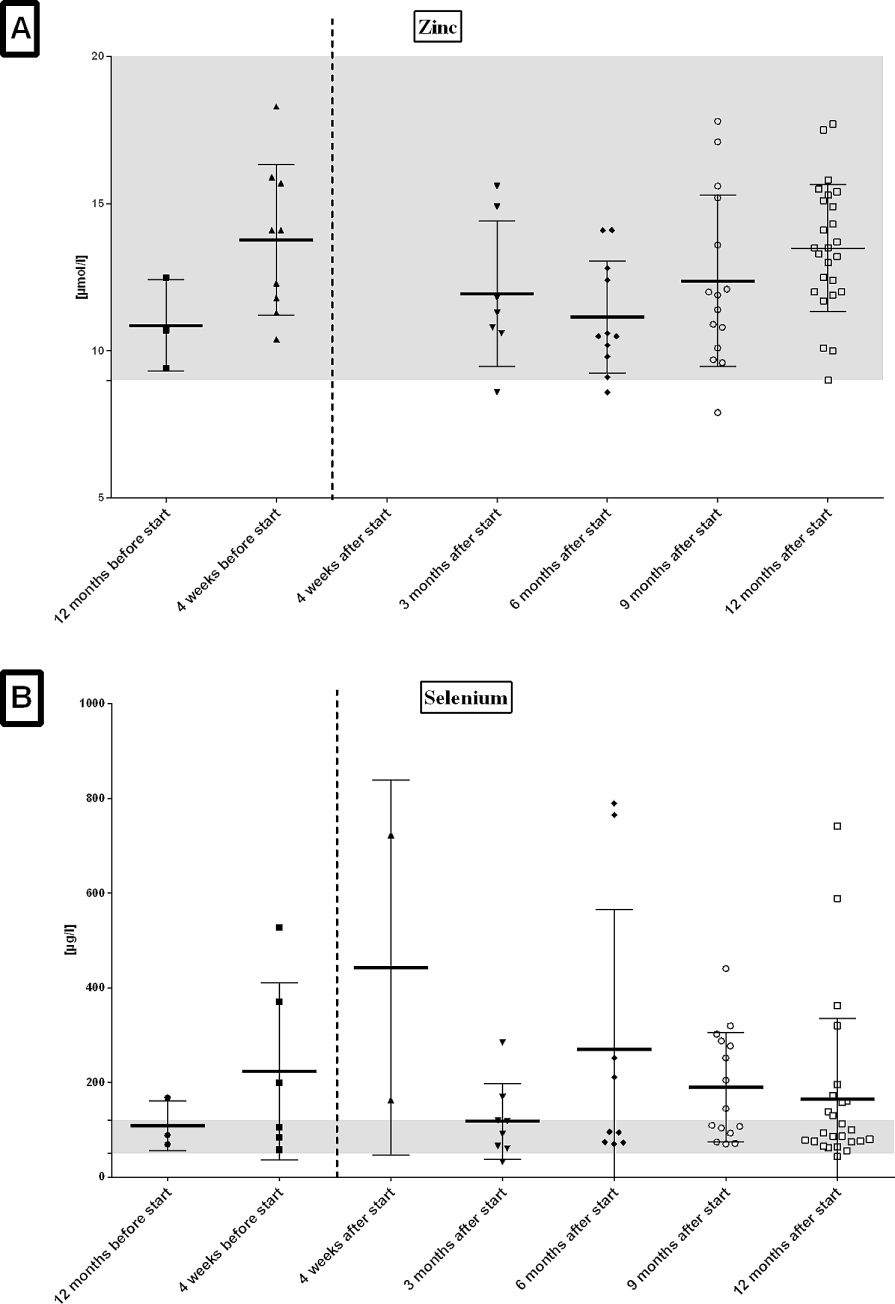
**A**: No changes in zinc plasma levels before and up to 12 months after start of ETI. **B**: No changes in selenium levels before and up to 12 months after start of ETI therapy in patients with cystic fibrosis. Missing data for zinc levels 4 weeks after start of ETI. Reference ranges of zinc (9–22 µmol/l) and selenium (50–120 µg/l) are indicated in grey. ETI = elexa-/teza-/ivacaftor.

**Fig. 3:**
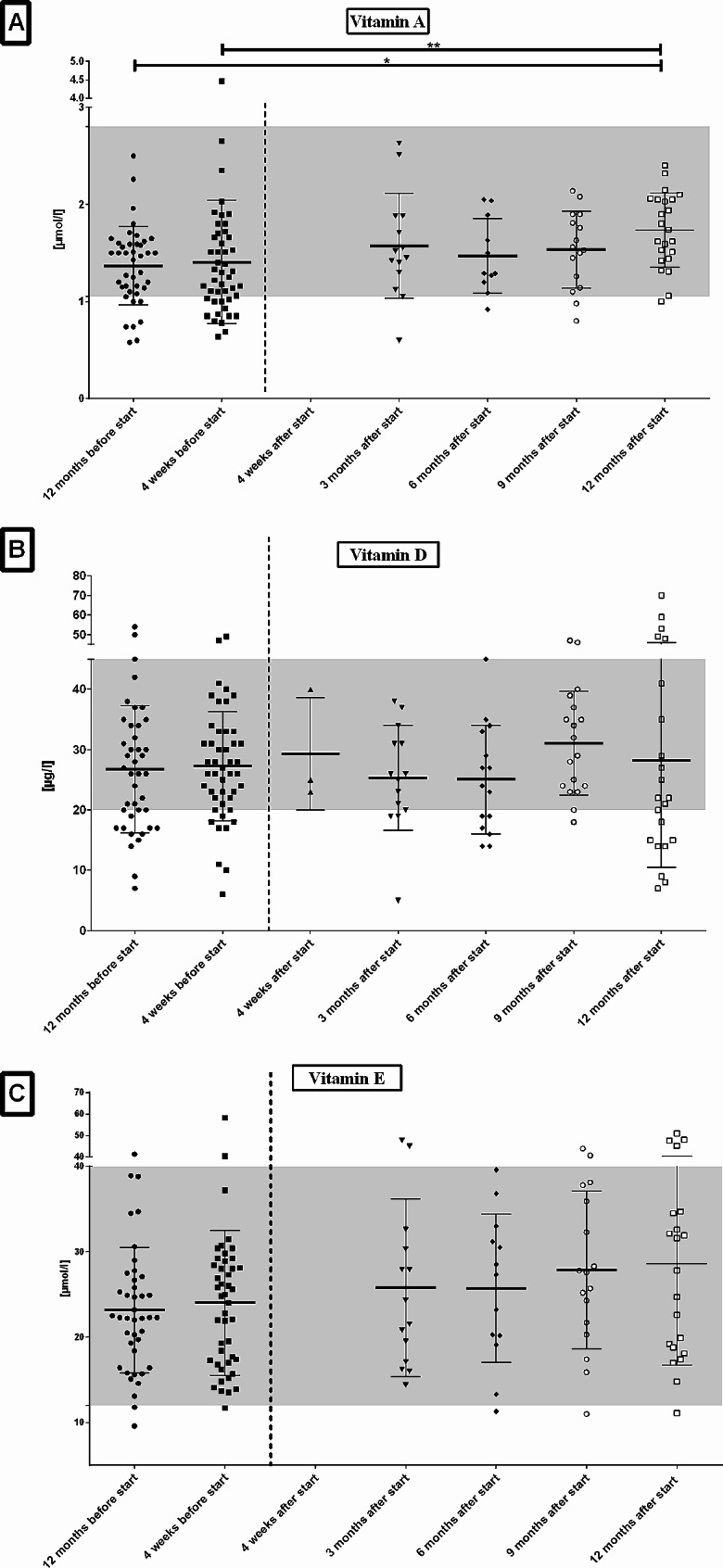
**A**: Significant increase of Vitamin A 12 months after start of ETI therapy in patients with cystic fibrosis. **B**: No changes in serum vitamin D levels and **C**: No changes in vitamin E levels over the study period after initiation of ETI therapy. Missing data at timepoint 4 weeks after start of ETI for vitamins A & E. Reference ranges of vitamin A (1.05–2.8 µmol/l), vitamin D (20–50 µg/l) and vitamin E (12–42 µmol/l) are indicated in grey. ETI = elexa-/teza-/ivacaftor.

After initiation of ETI patients showed a mild increase of AST that was not significant (Table 3; Fig. 1A). AST values normalized to similar levels as before during further course. Patients showed a significant increase of ALT four weeks after ETI start and a return to similar levels as before ETI in the following months (Fig. 1B). Bilirubin levels significantly increased after ETI and values remained significantly higher than before ETI during the follow-up (Fig. 1C). One patient was diagnosed with Gilbert’s syndrome due to persistent isolated elevation of bilirubin. Two further patients are suspected to have Gilbert’s syndrome but confirmation is pending. Four patients had to pause ETI therapy due to increased ALT/AST or bilirubin but all could re-start therapy and tolerated ETI well in further course. No further clinical abnormalities, for example abdominal pain, pruritus or icterus, were reported. No differences between both zinc and selenium levels at different time-points of follow-up were identified (Fig. 2A & B). Interestingly the most elevated selenium levels were seen in post-menopausal women. Vitamin A levels continuously rose during the study and the levels at 12 months’ follow-up were significantly higher than before ETI (Fig. 3A). Vitamin D levels showed a fluctuating course but no significant changes (Fig. 3B). Some patients showed persistent marked vitamin D deficiency even after starting ETI therapy and despite increased supplementation. Vitamin E values increased during the study but no significant change over the period of the study was noted (Fig. 3C). Sweat chloride fell significantly after ETI (*p* < .01) and values remained lower than before ETI during follow-up (Table 3).

No signs or symptoms of hypervitaminosis or vitamin-intoxication were found. Individuals with significantly elevated zinc, selenium, or vitamin levels received appropriately adjusted substitution therapy. Therefore, the observed changes in vitamin levels under ETI even could be understated because of adjusted substitution therapy. Previous therapy with luma-/ivacaftor in eight patients did not correlate with changes of any laboratory parameters or sweat chloride measurement after start of ETI.

There were no significant differences in laboratory parameters, sweat chloride or pulmonary function between children < 18 years and adults with cystic fibrosis. Homozygous or heterozygous genotype for Phe508del did not have an influence on any of the parameters as well. We did not observe differences over time depending on age or genotype in our cohort.

## Discussion

The data presented here show an improvement in both pulmonary function (measured in FEV1%pred) and sweat chloride in children and adults with cystic fibrosis homozygous and heterozygous for Phe508del treated with elexa-/teza-/ivacaftor. Changes in this heterogenous cohort are similar to previous reports regarding pulmonary function and sweat chloride [13, 16]. Neither age nor genotype did have an impact on laboratory parameters or pulmonary function in our study therefore only data of the whole cohort are reported. More evidence on extrapulmonary effects of ETI is emerging. This study shows effects on liver function tests, vitamin and trace element concentrations. In our cohort with an already normal BMI before ETI we did not find any significant change in weight as was found previously [13]. This discrepancy might be due to the heterogenous cohort including both children, pre-/postpubertal adolescents, and adults with CF.

ETI was well tolerated in our cohort. ETI therapy had to be paused briefly shortly after commencement of therapy in four individuals but all could continue ETI without significant side-effects. We found a significant increase of ALT four weeks after therapy initiation but levels returned to previous values in further course. Interestingly total bilirubin was permanently elevated after ETI but most patients still showed bilirubin values within the reference range. Increases of transaminases and bilirubin are well known side-effects of ETI therapy and only temporary in nature in most cases [13, 38, 39]. The data presented here show that most changes in transaminases or bilirubin were either transient in nature or values stayed in the normal ranges. Therefore, a change in patient care does not seem to be warranted but close follow-up is recommended. In one patient with persistent elevated indirect bilirubin Gilbert’s syndrome was diagnosed. Persistent isolated increase of bilirubin after ETI have demasked Gilbert’s syndrome in some cases after initiation of CFTR-modulator therapy [39, 40]. Liver enzymes and bilirubin are routinely evaluated after start of ETI. Persistent elevation of indirect bilirubin alone should lead to further diagnostics regarding Gilbert’s syndrome. Identification of Gilbert’s syndrome might allow patients to continue with ETI therapy despite bilirubin elevations even over the reference range.

In a mixed cohort of both children and adults with cystic fibrosis and different genotypes we found a significant increase of vitamin A 12 months after therapy with ETI and already normal values before therapy. Only one patient had elevated levels over 3.5 µmol/l before ETI therapy. No toxic effects were noted during the study period. Of note no patient showed pathologically increased vitamin A values, therefore supplementation of vitamin A was not changed in our cohort. Our cohort showed varying vitamin D levels and some patients still experienced vitamin D deficiency even after ETI therapy. No changes were found in vitamin D levels after initiation of ETI. Vitamin E levels rose continuously over the follow-up but the increase in vitamin E levels was not significant. The exact causes for improved uptake of vitamins and trace elements after initiation of CFTR-modulating therapy are not well understood. It has been suggested that cholesterol biosynthesis and uptake of cholesterol and fat-soluble micronutrients can be improved with CFTR-modulator therapy [22, 41, 42]. Reduced intestinal or pulmonary inflammation might contribute to improved vitamin levels as well [10, 43]. Additionally, patients might benefit from improved pancreatic function after start of CFTR-modulators [11, 44, 45] and this might influence uptake of vitamins and trace elements as well. Other reasons might include changes in intestinal pH, intestinal absorption of bile salts or changes in intestinal transit time [46]. Gaschignard et al. reported significantly increased Vitamin E levels 10 months after therapy with luma-/ivacaftor and Gelzo et al. reported higher vitamin E levels after luma-/ivacaftor therapy as well [21, 30]. Sommerburg et al. on the contrary reported a moderate but significant decrease in vitamin E and vitamin E/cholesterol one and two years after luma-/ivacaftor [22]. Vitamin A and D levels also rose, not-significantly in one study [30] but another report shows increased vitamin A levels [22]. Similar results have been published for ETI therapy: Both vitamins A and D levels increased in an adult cohort and vitamin A increased in a pediatric cohort [28, 41, 42]. A general improvement of fat-soluble vitamin levels has been reported as well after one year of ETI therapy in adults [23]. In some pediatric cases even hypervitaminosis A has been reported [47]. No changes in vitamin E and INR as an indirect measurement of vitamin K in adults and no changes in vitamin D and E levels in children were reported [41, 42]. Our data therefore are in accordance with the published literature regarding vitamin levels after start of highly-effective CFTR-modulator therapy with ETI. Slight differences in some vitamins could be explained by the different age structure of the respective cohorts and small patient numbers. In addition, measurements of vitamins did not occur at each study time point. A correlation of vitamin D levels with the season did not take place in our study. Even as fat-soluble vitamin deficiencies had been becoming less common before ETI therapy [22, 41] it seems worthwhile to follow-up individuals with cystic fibrosis more frequently regarding their vitamin status as an increase in levels of fat-soluble vitamins has been reported in most studies. The details regarding different vitamins differ between the studies but all come to the same conclusion: fat-soluble vitamin levels should be monitored more closely after ETI start than before. Evaluation of fat-soluble vitamins seems sensible after three, six, and 12 months of treatment and yearly afterwards. Regarding vitamin A it seems worthwhile to supplement with the lowest possible dose, especially in children. Data on vitamin E are less clear but no negative effects of increased vitamin E levels have been reported. Vitamin D seems to be affected the least and seasonal fluctuations must be considered.

The trace elements zinc and selenium were less frequently measured in the study presented here. Overall, no changes in these micronutrients were found before or after ETI therapy. Elevated levels for selenium were noted in post-menopausal females but no clinical significance could be determined. Similar considerations to those for improved vitamin absorption may apply to trace elements once ETI therapy is initiated. Zinc or selenium deficiency had become rare in CF even before ETI due to optimized supplementation. Especially the avoidance of zinc deficiency is associated with better outcomes in CF [32]. So far only one study showed an increase of selenium levels after therapy with lumacaftor/ivacaftor [30]. The study presented here is therefore the first to report outcomes regarding zinc and selenium levels after start of ETI therapy. Routine monitoring of trace elements is recommended and results need to be interpreted on a patient-individualized basis.

## What’s new about this study?

In the last two years, some evidence has been published that levels of fat-soluble vitamins increase after start of therapy with ETI in children and in adults and in different genotypes [21, 23, 28, 41, 42]. This study confirms these findings in a mixed cohort and adds to the evidence that neither age nor genotype seem to influence vitamin levels. This study is the first that examines trace element levels (zinc and selenium) after start of ETI. While the study is too small to draw generalizable conclusions, it is important to monitor trace element levels individually and adjust supplementation in some people with CF.

### Limitations

This is a retrospective monocentric study with a limited number of participants. The cohort consists of both children and adults with CF and includes both persons homozygous and heterozygous for Phe508del. Even though no differences in laboratory results were found depending on age or genotype the heterozygous study population has to be considered when interpreting results. Vitamins and trace elements were monitored not as frequently as other parameters and at some time-points only few laboratory values were included. Even though the study team tried to document all additional vitamin intake patients may have supplemented extra vitamins (e.g. over the counter preparations), and not informed their treating physicians in detail. Vitamin K, or at least coagulation values as surrogate parameters were not appreciated in this study, as vitamin K cannot be routinely monitored in our local laboratory and INR has been reported before to be an unreliable surrogate for vitamin K activity [22]. However, this study offers a real-world view on laboratory follow-up in children and adults with CF who started ETI.

## Conclusions

ETI therapy has been shown to significantly improve pulmonary function and quality of live in individuals with CF homozygous or heterozygous for Phe508del which could be reproduced in a mixed cohort of children (*n* = 14) and adults (*n* = 44) with CF. Close monitoring of liver function tests is warranted with some changes occurring rapidly and others more in the long term. All patients in our cohort tolerated ETI therapy in the long-term well and only four patients had to temporarily interrupt the therapy. Isolated persistent elevation of indirect bilirubin might be a sign for Gilbert’s syndrome. Fat-soluble vitamins and trace elements remain normal in most cases. It seems sensible to appreciate vitamin levels regularly after initiation of ETI therapy. Vitamin supplementation needs to be continued but individual compositions of vitamins and trace elements might be reasonable for personalized CF-therapy. Some vitamin levels seem to rise rather slowly so follow-up is necessary. Laboratory follow-up seems advisable after four weeks, three, six, nine and 12 months of the start of ETI and at least yearly afterwards. Even though most patients do not require a change in supplementation it is important to identify those few who do. Especially an elevated vitamin A can have severe side effects on patients. Whether changes in vitamins and trace elements are due to improved pancreatic function or other reasons, for example better intestinal absorption, should be the focus of future studies.

## Data Availability

Data is provided within the manuscript. Raw data are available upon reasonable request from the corresponding author.
